# Is DVT prophylaxis necessary after oncology lower limb surgery? A pilot study

**DOI:** 10.1186/s40064-016-2441-9

**Published:** 2016-06-30

**Authors:** Vivek Ajit Singh, Lim Ming Yong, Anushya Vijayananthan

**Affiliations:** Department of Orthopaedic Surgery, University of Malaya, 50603 Kuala Lumpur, Malaysia; Department of Biomedical Imaging, University of Malaya, 50603 Kuala Lumpur, Malaysia

## Abstract

**Background:**

Deep vein thrombosis (DVT) has been independently associated with both malignant diseases and orthopaedic surgery. Therefore, orthopaedic oncology patients may be at a high risk for thromboembolic events. However, less emphasis is given to this group of patients compared to the patients undergoing total hip and knee replacement. The objective of this study is to determine the incidence of DVT and their risk factors in patients undergoing orthopaedic oncology lower limb surgery without prophylaxis.

**Questions/purposes:**

(1) What is the incidence of DVT in patients who underwent orthopaedic oncology surgery for the lower limb? (2) What are the risk factors related to DVT in patients who underwent oncology surgery of the lower limb surgery? (3) This is a pilot study to determine if further trial is warranted.

**Methods:**

This is a prospective study. All sequential patients undergoing orthopaedic oncology operations from the period of 1st October 2013 till 30th September 2014 were recruited for the study with their consent. Their demographic data, diagnosis and surgery were documented. Thirty-eight patients who underwent lower limb surgeries for orthopaedic oncology indications were included in the study. No tourniquet was used in these lower limb surgeries. There were 24 men and 14 women with a mean age of 36 years (11–75). All potential risk factors were also identified and documented. All patients were not given any form of DVT prophylaxis (mechanical and chemical) before and after operation as this is a standard protocol in our center and a Medical Ethics Committee approval was taken for this study. DVT surveillance was performed 1 day before operation and 2 weeks after operation with ultrasound Doppler. Patients diagnosed with DVT via ultrasound Doppler were subsequently scheduled for CTPA to look for pulmonary embolism (PE).

**Results:**

DVT was detected in two patients (5 %). Both patients were asymptomatic and they both had proximal thrombosis. One patient (2.6 %) was diagnosed with non-fatal PE and was asymptomatic. PE was detected incidentally by staging computed tomography scan and the patient had negative ultrasound Doppler of the operated and non-operated limb for DVT. We did not carry out a statistical analysis as the study population with DVT and pulmonary embolism is small.

**Conclusions:**

The incidence of DVT in patients after undergoing orthopaedic oncology lower limb surgery was low even without prophylaxis at our center. Further investigation with larger sample size is needed to validate our results and identify the risk factors.

**Level of evidence:**

Level III descriptive study.

## Background

Deep vein thrombosis (DVT) is an important complication associated with patients with malignant disease who undergo orthopedic procedures. The incidence of DVT in patients undergoing hip fracture surgery alone comes with 40–60 % risk, according to literature (Geerts et al. 2004). Patients undergoing total joint replacement carry a 40–60 % risk of developing DVT without DVT prophylaxis (Cardiovascular Disease Educational and Research Trust and Cyprus Cardiovascular 2006; Geerts et al. 2004, 2008). Of the population which is newly diagnosed to have deep vein thrombosis, one-fifth of the patients diagnosed with DVT are oncology patients and the risk of a post-operative DVT is twice higher for oncology patients who undergo surgery, compared to patients without cancer while the risk of fatal pulmonary embolism (PE) is three fold higher for patients having malignant diseases compared to patients having surgery for benign conditions (Gallus and Hirsh 1976; Geerts et al. 2008; White et al. 2003). Furthermore, the incidence of DVT increased from six to seven folds among patients with malignancy, who received chemotherapy as part of their treatment compared to those with no malignancy (Heit et al. 2000). We conducted this pilot study to determine incidence of DVT in orthopaedic oncology patients who did not receive any form of DVT prophylaxis to see if further investigation is warranted.

In a prospective study done on cancer patients undergoing surgery, the authors look at 169 patients with cancer who underwent major oncological procedures of the lower limbs, they reported a 14.2 % risk of developing DVT and 0.6 % risk of developing PE (Lin et al. 1998). Few years later, the same group of researchers studied 87 consecutive patients who underwent hip replacement for oncologic indications and found that the rate of asymptomatic DVT and PE were 4 and 1.7 % respectively (Nathan et al. 2006). In both studies, all patients treated with intermittent pneumatic compression devices and in the second study, majority of the patients received anticoagulants. That would probably explain the lower rate of DVT. Furthermore, the patient population in the first study was varied. According to other studies, there is a 21 % (Tuy et al. 2009) up to 22 % (Robinson et al. 1998) risk of developing DVT among patients undergoing oncology surgery of the lower limb. There are a few retrospective studies on the incidence of symptomatic venous thromboembolism after orthopedic surgeries in oncology patients. These studies reported that the rate of DVT and PE among these patients ranged from 1.1 to 5 % and 0.6 to 2.3 % respectively (Jeys et al. 2008; Kim et al. 2013; Mitchell et al. 2007; Morii et al. 2010; Patel et al. 2010; Ramo et al. 2011; Ruggieri et al. 2010).

The association between occult or overt malignancy and thrombosis has been widely accepted since Trousseau in 1865. Virchow’s triad describes three broad categories of factors contributing to the development of thrombosis, namely hypercoagulability state, hemodynamic changes and endothelial injury or dysfunction. Oncology patients have a few major risk factors predisposing them to DVT and PE. These factors may be classified into tumour-related, patient-related or treatment-related. Tumour-related factors include tumour histology, tumour location, stage of disease, and duration of disease. Risk of developing DVT was reported to be higher among patients diagnosed with malignant disease compared to those with benign tumor (Morii et al. 2010). Moreover, the risk of developing DVT among oncology patients increased by twofold with the presence of metastasis (Sood 2009).

Patient-related factors include advanced age, female gender, pregnancy, and previous history of DVT, the presence of co-morbidity and hypercoagulable states. Malignancy could also lead to hypercoagulopathy (Morii et al. 2010). There are a few reasons predisposing oncology patients to hypercoagulability state. First is the interaction between the tumor cells and monocytes and macrophages, causing the release of chemical mediators such as IL-1, IL-6 and TNF. These chemical mediators would cause endothelial injury and also lead to thrombus formation. Second factor is the activation of coagulation pathway by the expression of tissue factor that has undergone malignant transformation. This would lead to the activation of the coagulation cascade through the extrinsic pathway.

Treatment-related factors include duration of hospitalization, pharmacologic measures taken and mechanical interventions. Pharmacologic measures consist of chemotherapeutic agents, hormonal agents and anti-angiogenic agents whereas mechanical interventions include surgery and introduction of central venous catheters. Chemotherapy increases the risk of venous-thromboembolism (VTE) by approximately six to seven folds in patients with cancer compared to patients without cancer (Silverstein et al. 1998). Chemotherapeutic drugs may also cause damage to the vascular endothelium and activate platelets aggregation. Furthermore, they also facilitate induction of tissue factor in tumour cells and down regulate anticoagulant proteins such as protein C and S.

### Rationale

The literature on the need for the use of DVT prophylaxis is orthopaedic oncology patients going for surgery is unclear and confusing, therefore there is a need for a prospective study to define the risk of VTE and it’s risk factors. The aim of this study is to determine the incidence of deep vein thrombosis in patients who underwent oncology lower limb surgeries and the associated risk factors. Thus, to determine the need for the use of DVT prophylaxis in these patients.

### Study questions

What is the incidence of DVT in patients who underwent orthopaedic oncology surgery for the lower limb?

What are the risk factors related to DVT in patients who underwent oncology surgery of the lower limb surgery?

## Methods

### Study design and setting

This study was carried out in the Orthopedic Oncology Unit, University Malaya Medical Center, Kuala Lumpur. It was a prospective study and carried out from 1st October 2013 to 30th September 2014. Informed consent was obtained for the study and the Ultrasound Doppler and Computed Tomographic Pulmonary Angiography (CTPA) when needed. The study protocol was approved by the Medical Ethics Committee University Malaya Medical Center (Reference number: 1010.20).

### Participants/study subjects

Inclusion criteria:

1. Patients undergoing major lower limb oncology surgery: wide excision of tumour, limb salvage surgery with endoprosthetic replacement, long stem total hip replacement and hinge knee replacement.
2. Patients diagnosed with either malignant or benign tumour.

Exclusion criteria:

1. Minor surgeries: Example: Biopsy, wound debridement, lengthening of endoprosthesis, Manipulation Under Anesthesia.
2. Amputation: Hemipelvectomy, above or below knee amputation, trans-metatarsal amputation, ray’s amputation or disarticulation of toes.
3. Patients who were given chemoprophylaxis for deep vein thrombosis.
4. Failure to obtain consent.

### Randomization

All sequential patients who qualified according to the inclusion criteria were included.

### Description of experiment, treatment, or surgery

For each patient a complete data was documented including demographic details, diagnosis on admission, the nature and duration of surgery, any chemotherapy undergone and any risk factors available. The risk factors, which we recorded, include previous history of DVT or PE, age more than 40 years old, prolong immobility, thrombophilia, chronic lung disease, ischaemic stroke, congestive heart disease, obesity, hormonal therapy and admission to intensive care unit. All patients did not receive any mechanical or chemoprophylaxis for DVT as this is the routine practice at our center.

Informed consent was obtained and patients were subjected to bilateral lower limb ultrasound Doppler done 1 day before the operation, to look for any pre-existing deep vein thrombosis. After operation, patients were observed closely for any signs of DVT or pulmonary embolism. Bilateral lower limb ultrasound Doppler screening for DVT was repeated at 14 days post-operatively. Fourteen days was chosen because all the subjects were fully ambulant by then. If there was suspicion of clinical DVT or pulmonary embolism, ultrasound Doppler was performed earlier than the proposed 14 days post-operatively. The ultrasound Doppler was performed by a designated ultrasonographer and reported by a radiologist. Any evidence of DVT detected on the calf was classified as distal and those at popliteal and proximal to it were classified as proximal.

### Aftercare

Patients who were found to have DVT subjected to computed tomography pulmonary angiography (CTPA) to look for evidence of pulmonary embolism and were treated with warfarin for 3–6 months.

### Description of follow up routine

All patients were reviewed 2 weeks after the surgery and examined both clinically and Ultrasound Doppler repeated to look for evidence of DVT.

### Variables, outcome measures, data sources, and bias

Ultrasound Doppler was performed with the patients in supine position. Ultrasound Doppler was performed using a Philips iU22 ultrasound machine. The criteria for positive result for DVT include the direct visualization of thrombus, the absence of spontaneous flow by Doppler, absence of phasicity of flow with respiration, and the incompressibility of deep veins with probe pressure. During the compression test, the ultrasonographer pressed the transducer to the skin overlying the vein with enough force to cause the walls of the vessel to collapse, obliterating the lumen. Inability to compress the vein completely was considered diagnostic indicator for the presence of a thrombus. In addition, while the patient performed a Valsalva maneuver, the ultrasonographer observed the lumen of the vein. Dilation of the vein was an indicator for the absence of a thrombus. The result was considered to be negative if the lumen of the proximal veins could be completely occluded with compression. Positive findings at calf region were considered as distal DVT whereas positive findings at popliteal region or proximal to it were considered proximal DVT.

During CTPA, a high resolution computed tomography of the entire thorax was done first. The initial scan was performed without contrast to detect any alternative findings pertaining to patients’ symptoms. Subsequent imaging was performed with injection of contrast via a large bore branula (at least size 18 Gauge). Findings were considered positive for pulmonary embolism if an intra-luminal filling defect was seen within a pulmonary arterial vessel and was considered negative if no filling defect was observed. The results were only considered technically inadequate if the lobar or main pulmonary vessels were not visualized.

### Statistical analysis, study size

A total of 62 patients underwent orthopaedic oncology operations during the study period and only 38 patients fit the inclusion criteria and were included in this study. The sample size was not determined statistically. Statistical analysis of the results was not performed, as the study population with DVT and pulmonary embolism was small.

### Demographics, description of study population

The study population consists of all patient undergoing resection of lower limb tumours for oncological reasons.

### Accounting for all patients/study subjects

We had a total of 38 patients recruited and they are all still under our follow up. There is no patients’ loss to follow up.

## Results

What is the incidence of DVT in patients who underwent orthopaedic oncology surgery for the lower limb?

The study population consists of 24 males and 14 females. Their mean age of our patients was 36 years (11–75). Their histological diagnosis is summarized in Table 1. Eighty-nine percent of patients (34 of 38) were diagnosed with malignant tumour and 11 % (4 of 38) with benign tumour. The most common histological diagnosis was sarcoma (29 out of 38) comprising 76 % of the patients in this study. There were 21 patients with soft tissue sarcoma and 8 patients with osteosarcoma. Most patients presented with tumor situated in pelvis, hip or thigh [22 patients (58 %)] and 16 patients (42 %) had tumor located in knee, tibia/fibula or foot. There were equal numbers of patients with bone versus soft tissue as origin of tumour. Out of 38 patients, 27 patients (71 %) received chemotherapy as part of their treatment.

**Table 1:** Histological diagnosis

Type of tumor	Number of patients
*Cancer type*	
Sarcoma	29
Osteosarcoma	8
Soft tissue sarcoma	21
Malignant melanoma	1
Flexiform neurofibroma	1
Lymphangioma	1
Chondroblastoma	1
Osteochondroma	1
Bone metastasis	1

The most common surgical procedure carried out was wide resection, which was performed in 36 of the patients (95 %) (Table 2). Of the 36 cases of wide resection, 16 involved reconstruction with endoprosthesis (44 %). Other procedures include total hip replacement (1 out of 38), and curettage and bone graft (1 out of 38). The average duration of surgery was 78 min (range 30–170 min). In most patients [29 out of 38 patients (81 %)], the surgery was carried out in less than 2 h.

**Table 2:** Type of surgery

Type of surgery	Number of patients
Wide excision	36
With reconstructive procedure	16
Without reconstructive procedure	20
Curettage and bone graft	1
Total hip replacement	1

The average duration of immobilization in the post-operative period among the study subjects was 2 days (range 1–5 days). For patients who underwent surgery without reconstruction, average period of immobilization post-operative was 1 day (range 1–2 days). For patients who underwent reconstruction surgery, the average period of immobilization was 4 days (range 3–5 days).

From the 38 patients in the study population, the rate of DVT detected post-operatively was 5 % (2 of 38). Both patients were asymptomatic of DVT. In both cases, the location of thrombosis detected was proximal and on the same side as the tumour. Both patients were aged less than 40 year old. One patient was diagnosed with osteosarcoma of proximal right tibia, in which wide resection with reconstruction surgery was performed. She received neoadjuvant chemotherapy as part of her treatment. The other patient was diagnosed with localize plexiform neurofibroma of right thigh and wide resection was performed. There was no pulmonary embolism detected by CTPA on these two patients and they received warfarin as treatment of DVT for 6 months. Their details were summarized in Table 3.

**Table 3:** Details of patients with DVT and PE

Pt	Sex	Age	Diagnosis	Procedure	Duration of surgery (min)	Risk factors	DVT	PE
1	F	16	Osteosarcoma	Endo-prosthesis	135	Chemotherapy	Proximal	No
2	M	31	Plexiform neurofibroma	Wide excision	60	No	Proximal	No
3	F	52	Soft tissue sarcoma	Wide excision	60	Age > 40	No	Yes

All cases of deep vein thrombosis and pulmonary embolism in the study

**Fig. 1:**
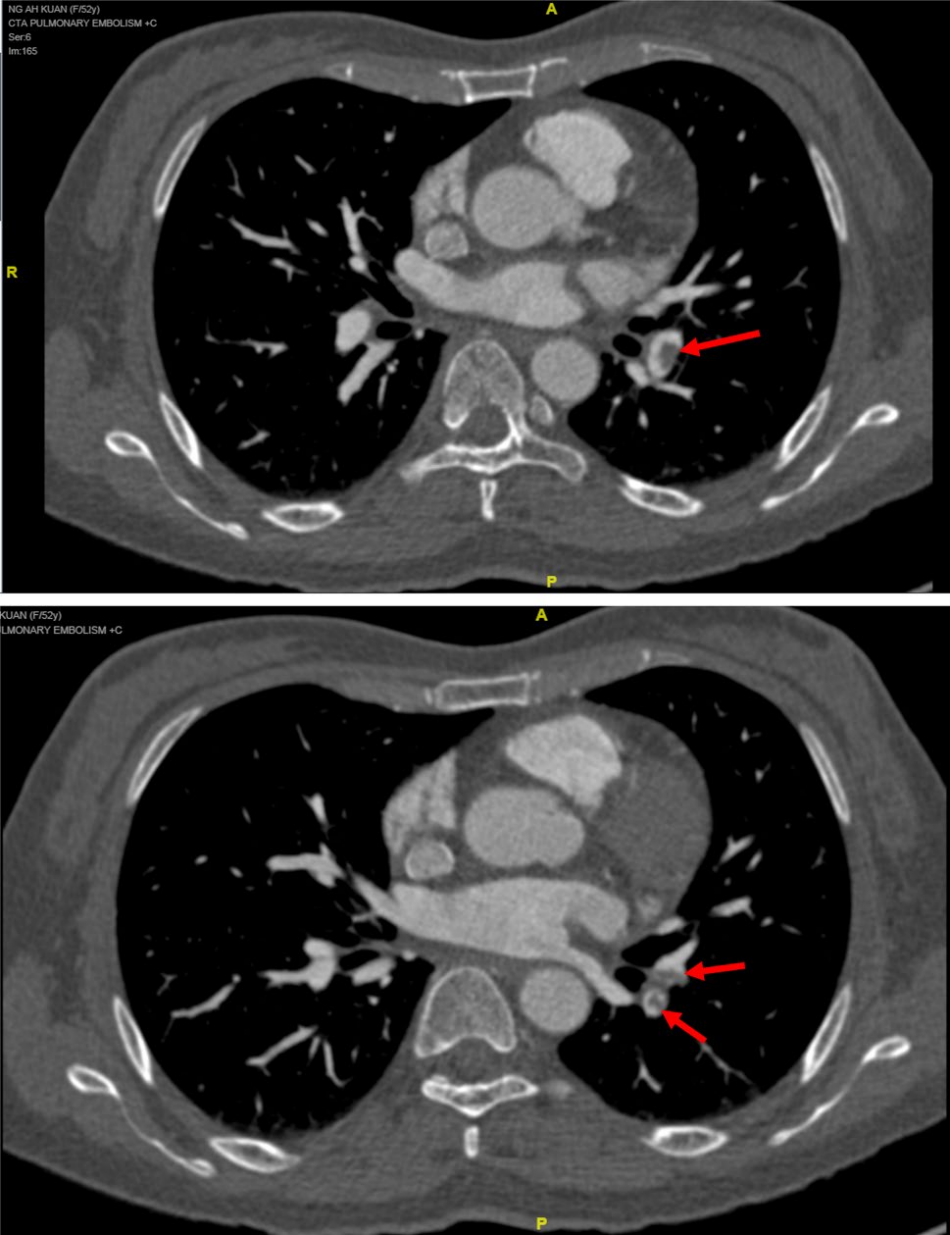
CTPA showing filling defect in left descending pulmonary artery extending to segmental branch

There was only one patient in the study population who was found to have pulmonary embolism (PE). She was diagnosed with soft tissue sarcoma of right thigh and wide resection was performed. The PE was detected incidentally by a scheduled CT Thorax-Abdomen-Pelvis (CT TAP) which was carried out as part of her post-operative staging during the 3rd week after the operation. The diagnosis was then confirmed by CTPA (Fig. 1). There was filling defect detected in the left descending pulmonary artery extending to the segmental branches. She was asymptomatic of PE. Ultrasound Doppler done for this patient showed no evidence of DVT bilaterally. No VTE-related lethal event was reported during the study period.

What are the risk factors related to DVT in patients who underwent oncology surgery of the lower limb surgery?

The risk factors for DVT are shown in Table 4. A statistical analysis was not performed as the study population with DVT and pulmonary embolism was small.

**Table 4:** Risk factors in patients with and without DVT

Category	Number of patients	DVT	No DVT
Total	38	2	36
Age			
≤40	22	2	20
>40	16	0	16
Gender			
Male	24	1	23
Female	14	1	13
Tumor histology			
Benign	4	1	3
Malignant	34	1	33
Tumor location			
Proximal	22	1	21
Distal	16	1	15
Tumor origin			
Bone	19	1	18
Soft tissue	19	1	18
Reconstructive surgery			
Yes	17	1	16
No	21	1	20
Operation time (min)			
<120	29	1	28
≥120	9	1	8
Chemotherapy			
Yes	27	1	26
No	11	1	10

## Discussion

### Background and rationale

The main goal of this study was to determine the rate of deep vein thrombosis in patients underwent oncology lower limb surgeries and the need for routine DVT prophylaxis.

### Discussion: What is the incidence of DVT in patients who underwent orthopaedic oncology surgery for the lower limb?

Without the use of prophylaxis, the rate of DVT in this study was 5 % and only one patient from the study population developed PE. Of these, none of the patients were symptomatic and the single case of PE was detected incidentally using a CT scan. There was no fatal PE reported.

There are few factors that should be taken into consideration for this study. Firstly, both benign and malignant cases were included in this study. Secondly, no mechanical or chemoprophylaxis was given to the patients before and after operation. Last but not least, all the patients were screened for DVT by an experienced ultrasonographer before and after the operation. Therefore, the precise incidence of DVT after oncology lower limb surgeries was identified.

There are currently limited numbers of prospective studies addressing the incidence of DVT in patients after oncology lower limb surgery (Table 5). However, it is difficult to compare the incidence of DVT from literature due to the variations in characteristics of patients, type of prophylaxis and method of DVT detection differs between the different studies. Lin et al. (1998) reported a 14.2 % incidence of DVT and 0.6 % incidence of PE after orthopaedic surgery in adult cancer patients. Tuy et al. (2009) reported a 21 % incidence of DVT and 2 % of PE in patients underwent musculoskeletal tumour surgery involving pelvic or lower limb malignancy. Recently, Yamaguchi et al. (2013) reported a 22 % incidence of DVT and 1 % incidence of PE after resection of musculoskeletal tumours of lower limb. All the patients from these studies were only given mechanical prophylaxis (intermittent compression device) and not chemoprophylaxis. IVC filter was used to prevent PE in the study by Tuy et al. (2009), Ultrasound Doppler was used as the method for detection of DVT in these studies. These reported incidences were substantially higher compared to another prospective study by Nathan et al. (2006), which reported a 4 % incidence of DVT and 1.1 % of PE after hip replacement for oncologic indications. All of the patients in their study received intermittent compression device. The difference between this study and others is that most of the patients were given low-molecular-weight-heparin (LMWH) as a prevention of DVT. Only 9 out of 87 of patients did not receive LMWH due to high risk of hemorrhage. Even without mechanical prophylaxis, the incidence of DVT and PE in this study was comparable to the report by Nathan et al., This may be due to early mobilization of the patients post-operatively, which is evident by the average period of immobilization of 2 days in this study.

**Table 5:** Summary of previous prospective studies on incidence of DVT

Author	Study sample	VTE prophylaxis		DVT (%)	PE (%)
		Mechanical	Chemoprophylaxis		
Lin et al. (1998)	Malignant musculoskeletal tumor	Yes	No	14.2	0.6
Nathan et al. (2006)	Malignant musculoskeletal tumor	Yes	Yes (most patients)	4	1.1
Tuy et al. (2009)	Malignant musculoskeletal tumor	Yes	No	21	2
Yamaguchi et al. (2013)	Benign and malignant musculoskeletal tumor	Yes	No	22	1
This study	Benign and malignant musculoskeletal tumor	No	No	5	2.6

There are a few retrospective studies addressing the incidence of DVT in patients after oncology lower limb surgery. In these studies, only the rate of clinically apparent symptomatic PE was reported. Kim et al. (2013) reported a 4.8 % incidence of DVT and 0.6 % incidence of fatal PE in 168 patients with malignancy of lower limb, post-operatively. Mitchell et al. (2007) reported a 4 % incidence of DVT and 1.2 % incidence of PE in patients with primary bone or soft tissue sarcoma. Patel et al. (2010) reported a 5 % incidence of DVT and 1.1 % incidence of PE in 348 oncology patients undergoing orthopaedic procedures for musculoskeletal neoplasm of the pelvis or lower extremity. Benevenia et al. (2004) reported an 8 % incidence of DVT and 0 % incidence of PE in patients with metastatic pathologic fractures of the lower extremity. In this study, the incidence of DVT was comparable to Kim et al., Mitchell et al., Patel et al., and Benevenia et al., even though our patients were asymptomatic. There are few retrospective studies reporting a lower incidence of DVT in patients who underwent oncology lower limb surgery (Heit et al. 2000; Mitchell et al. 2007; Ramo et al. 2011; Ruggieri et al. 2010). The incidence ranged from 1.1 to 2.4 % for DVT and 0.1 to 2.3 % for PE. Heit et al. did a cohort study of 625 patients from various groups including those who underwent surgery, those in hospital or nursing homes, trauma patients and oncology patients. He concluded that hospital or nursing home confinement, surgery, trauma, malignant neoplasm, chemotherapy, neurologic disease with paresis, central venous catheter or pacemaker, varicose veins, and superficial vein thrombosis are independent and important risk factors for VTE (Heit et al. 2000). Mitchell et al. (2007) looked at 252 patients with bone and soft tissue sarcomas who underwent surgery and concluded that risk of a clinically apparent thromboembolic event in patients with bone or soft-tissue sarcomas is comparable with that in other orthopaedic patients. Ramo et al. (2011) studied the incidence of DVT in 423 patients who underwent endoprosthesis replacement and he concluded that the incidence of symptomatic venous thromboembolism in their group of cancer patients who underwent lower-extremity endoprosthetic arthroplasty was lower than anticipated. Ruggieri et al. (2010) looked at his population of 986 patients with uncemented endoprosthesis. He concluded that the clinical occurrence of this DVT was extremely low probably due to a consistent and careful prophylaxis, prolonged until the time of complete weight-bearing.

Without prophylaxis, the incidence of DVT in total joint arthroplasty patients ranged from 40 to 80 % (Cardiovascular Disease Educational and Research Trust and Cyprus Cardiovascular 2006; Geerts et al. 2004; Murray et al. 1996; Sood 2009). Our reported DVT incidence of 5 % among patients who underwent oncology lower limb surgery without prophylaxis was substantially lower. Our result also showed lower incidence of DVT compared to patients undergoing hip fracture surgeries, which carries a risk of 40–60 % (Geerts et al. 2004).

There is no clear guideline currently available on DVT prophylaxis for patients undergoing oncology lower limb surgery. The American Society of Clinical Oncology (ASCO) developed recommendations for prophylaxis among oncology patients undergoing major surgical operations which mainly consists of abdominal and pelvis surgeries (Kuderer and Lyman 2014). Whereas in the latest American College of Chest Physicians (ACCP) guideline, there is recommendation only for the prophylaxis of cancer patients in out-patient settings (Guyatt et al. 2012). There are not committed on the use of chemoprophylaxis in this group of patients. Kim et al. (2013) and Ramo et al. (2011) reported that there is no statistical difference between patients given chemoprophylaxis and those who were not given chemoprophylaxis in the occurrence of DVT after oncology lower limb surgeries. On the other hand, Nathan et al. (2006), and Yamaguchi et al. (2013) stated that the incidence of DVT is high when only mechanical prophylaxis is used. Therefore, they suggested a combination of mechanical and chemoprophylaxis in this group of patients to effectively reduce the rate of DVT. Our study suggests that there is no pressing need for the use of either mechanical or chemoprophylaxis in patients undergoing lower limb surgery for orthopaedic oncology.

### Discussion: What are the risk factors related to DVT in patients who underwent oncology surgery of the lower limb surgery?

There are few studies addressing the association between the occurrence of DVT and risk factors after oncology lower limb surgeries. Nathan et al. (2006) reported higher incidence of DVT among patients with sarcoma as well as tumour located in the pelvis. Likewise, Mitchell et al. (2007) also reported that tumours in the thigh and hip regions are associated with higher incidence of DVT. Age was reported as an associated factor to the development of DVT by Yamaguchi et al. (2013) and Kim et al. (2013). In addition, Kim et al. also reported a statistically significant association between the occurrence of DVT and metastatic tumour. As for the type of surgery as a risk factor, Tuy et al. (2009) reported higher incidence of DVT among patients who underwent reconstructive surgery. The association between chemotherapy and DVT was only found by Morii et al. (2010) to be statistically significant. Lin et al. (1998) and Ramo et al. (2011) found no association between the occurrence of DVT and risk factors. The risk factors associated with DVT and PE were not identified as the study population and incidence rates in this study are small.

### Limitations

Firstly, as this is a pilot study, the number of patients or study subjects was small. The small sample size limits the conclusions that may be drawn and some predictive variables may not have sufficient statistical significance. A further trial in necessary to determine the impact of not using DVT prophylaxis in orthopaedic oncology patients undergoing surgery. Secondly, the ultrasound Doppler was used as method for DVT detection. Although ultrasound Doppler is considered to be effective and non-invasive, but it is still inferior compared to contrast venography. Ultrasound Doppler has been proven to be effective for detection of proximal thrombosis in patients after orthopaedic surgery but is less accurate to detect distal DVT (Robinson et al. 1998; Schellong et al. 2007). Furthermore, Ultrasound Doppler is operator dependent and this can lead to false negative results. Thirdly, routine CTPA was not performed in this study. Therefore, asymptomatic PE without the presence of lower limb DVT may have been missed. Lastly, the patient population was heterogeneous and it included patients under the age of 18 years old. There was also a wide variation in the histology diagnosis, location and type of surgeries done.

## Conclusions

In summary, this study demonstrates the incidence of DVT in patients after undergoing lower limb orthopaedic oncology surgery was low even without the use of mechanical or chemoprophylaxis in our center. The incidence of DVT and PE was not as high as anticipated. Further investigation with larger sample sizes would be useful to validate this result and identify the risk factors.
